# Microglial-derived microparticles mediate neuroinflammation after traumatic brain injury

**DOI:** 10.1186/s12974-017-0819-4

**Published:** 2017-03-15

**Authors:** Alok Kumar, Bogdan A. Stoica, David J. Loane, Ming Yang, Gelareh Abulwerdi, Niaz Khan, Asit Kumar, Stephen R. Thom, Alan I. Faden

**Affiliations:** 1 Department of Anesthesiology, University of Maryland School of Medicine, Baltimore, MD USA; 2 Shock, Trauma and Anesthesiology Research (STAR) Center, University of Maryland School of Medicine, Health Sciences Facility II (HSFII), #S247 20 Penn Street, Baltimore, MD 21201 USA; 3 Department of Emergency Medicine, University of Maryland School of Medicine, Baltimore, MD USA

**Keywords:** Microparticles, Microglia, Neuroinflammation, Traumatic brain injury, Interleukin-1β, miR-155

## Abstract

**Background:**

Local and systemic inflammatory responses are initiated early after traumatic brain injury (TBI), and may play a key role in the secondary injury processes resulting in neuronal loss and neurological deficits. However, the mechanisms responsible for the rapid expansion of neuroinflammation and its long-term progression have yet to be elucidated. Here, we investigate the role of microparticles (MP), a member of the extracellular vesicle family, in the exchange of pro-inflammatory molecules between brain immune cells, as well as their transfer to the systemic circulation, as key pathways of inflammation propagation following brain trauma.

**Methods:**

Adult male C57BL/6 mice were subjected to controlled cortical impact TBI for 24 h, and enriched MP were isolated in the blood, while neuroinflammation was assessed in the TBI cortex. MP were characterized by flow cytometry, and MP content was assayed using gene and protein markers for pro-inflammatory mediators. Enriched MP co-cultured with BV2 or primary microglial cells were used for immune propagation assays. Enriched MP from BV2 microglia or CD11b-positive microglia from the TBI brain were stereotactically injected into the cortex of uninjured mice to evaluate MP-related seeding of neuroinflammation in vivo.

**Results:**

As the neuroinflammatory response is developing in the brain after TBI, microglial-derived MP are released into the circulation. Circulating enriched MP from the TBI animals can activate microglia in vitro. Lipopolysaccharide stimulation increases MP release from microglia in vitro and enhances their content of pro-inflammatory mediators, interleukin-1β and microRNA-155. Enriched MP from activated microglia in vitro or CD11b-isolated microglia/macrophage from the TBI brain ex vivo are sufficient to initiate neuroinflammation following their injection into the cortex of naïve (uninjured) animals.

**Conclusions:**

These data provide further insights into the mechanisms underlying the development and dissemination of neuroinflammation after TBI. MP loaded with pro-inflammatory molecules initially released by microglia following trauma can activate additional microglia that may contribute to progressive neuroinflammatory response in the injured brain, as well as stimulate systemic immune responses. Due to their ability to independently initiate inflammatory responses, MP derived from activated microglia may provide a potential therapeutic target for other neurological disorders in which neuroinflammation may be a contributing factor.

## Background

Microparticles (MP; also called microvesicles), a type of extracellular vesicle, are small membrane-bound bodies shed from the plasma membrane and released by cells during activation or cell death [1]. They are composed of the plasma membrane along with a limited amount of cytoplasm and measure 100 to 1000 nm in diameter [2]. MP are enriched in the lipid microdomains, where cholesterol, phospholipids, and receptors are clustered [3], and are distinguished from other extracellular vesicles such as exosomes (30–100 nm; endosomal origin) and apoptotic bodies (1000–2000 nm) [1]. MP can be released from virtually all cell types in the brain and contain molecular signals in the form of non-secreted proteins and DNA/RNA/microRNA (miR) molecules that may be involved in cell-to-cell communication during neurodevelopment and synaptic physiology [4]. MP and other extracellular vesicles have also been implicated in the development and progression of various neurological diseases [4]. For example, in transmissible spongiform encephalopathy accumulation and cell-to-cell transmission of infectious prion proteins (PrP^sc^), extracellular vesicles are a key mechanism of prion disease propagation [5, 6]. In amyotrophic lateral sclerosis, accumulating evidence indicates that a mechanism for the progressive accumulation of misfolded mutant form of superoxide dismutase 1 (SOD1) is cell-to-cell propagation of SOD1 within the brain via extracellular vesicles that extend the range and toxicity during disease [7, 8]. Similar mechanisms are thought to contribute to neurotoxic amyloid-β formation in Alzheimer’s disease models [9–11]; however, these data are more controversial given that other studies have shown that exosome-associated amyloid-β are neuroprotective [12, 13].

Traumatic brain injury (TBI) causes cell death and neurologic dysfunction through secondary injury mechanisms characterized by edema, neuronal cell death, glial activation, and infiltration of peripheral immune cells, among others [14]. The inflammatory response to TBI is highly complex and includes rapid proliferation and migration of resident microglia to the site of injury in response to damage-associated molecular patterns (DAMPs) and other factors released by injured tissue [15], as well as by infiltration of neutrophils and inflammatory monocyte subsets that contribute to the injury milieu [16]. If the immune response is unable to resolve effectively, or becomes dysregulated after TBI, it can contribute to chronic neurodegeneration, due to chronic activation of neurotoxic microglia [17]. Elevated levels of MP have been reported in the cerebrospinal fluid of TBI patients [18], and circulating MP derived from endothelial cells, platelets, and leukocytes have been measured acutely after severe isolated TBI [19]. These include cerebral endothelium-derived MP release following focal contusion injury [20] as well as neuronal- and astroglial-derived MP release following mixed contusion and diffuse axonal injury [21].

Given the increasingly recognized role for neuroinflammation in tissue damage after TBI, we examined whether posttraumatic circulating MP released after injury could be derived from microglia, as well as potential mechanisms of microglial MP involvement in cell-to-cell communication in the brain following trauma. We hypothesized that MP released by microglia contain pro-inflammatory molecules that contribute to the spread of brain inflammation. We used flow cytometry to identify the origin of brain-specific immune-related MP in the circulation following controlled cortical impact in mice. To investigate immunogenic properties of microglial-derived MP, we used BV2 microglia and primary microglial cell culture models. Finally, to demonstrate cell-to-cell propagation of neuroinflammatory mechanisms within the brain, we injected enriched MP derived from microglia isolated from TBI cortex or lipopolysaccharide (LPS)-stimulated microglia into the cortex of uninjured mice.

## Methods

### Animals

Studies were performed using C57BL/6 adult male mice (10–12 weeks old, 22–26 g). The mice were housed in the Animal Care facility at the University of Maryland School of Medicine under a 12-h light-dark cycle with ad libitum access to food and water. All surgical procedures were carried out in accordance with protocols approved by the Institutional Animal Care and Use Committee (IACUC) at the University of Maryland School of Medicine.

### Controlled cortical impact

Our custom-designed controlled cortical impact (CCI) TBI device consists of a microprocessor-controlled pneumatic impactor with a 3.5-mm diameter tip. Mice were anesthetized with isoflurane evaporated in a gas mixture containing 70% N_2_O and 30% O_2_ and administered through a nose mask (induction at 4% and maintenance at 2%). Depth of anesthesia was assessed by monitoring respiration rate and pedal withdrawal reflexes. Mice were placed on a heated pad, and core body temperature was maintained at 37 °C. The head was mounted in a stereotaxic frame, and the surgical site was clipped and cleaned with Nolvasan and ethanol scrubs. A 10-mm midline incision was made over the skull, the skin and fascia were reflected, and a 5-mm craniotomy was made on the central aspect of the left parietal bone. The impounder tip of the injury device was then extended to its full stroke distance (44 mm), positioned to the surface of the exposed dura, and reset to impact the cortical surface. Moderate-level TBI was induced using an impactor velocity of 6 m/s and deformation depth of 2 mm as previously described [22]. After injury, the incision was closed with interrupted 6-0 silk sutures, anesthesia was terminated, and the animal was placed into a heated cage to maintain normal core temperature for 45 min post-injury. Sham animals underwent the same procedure as TBI mice except for the impact.

#### Study 1

Sham (*n* = 6) and TBI (*n* = 6) of C57BL/6J mice were anesthetized (100 mg/kg sodium pentobarbital, I.P.) at 24 h post-injury, and blood was collected in heparinized syringes by aortic puncture for blood MP analysis. Ipsilateral cortical tissue was rapidly dissected and snap-frozen on liquid nitrogen for RNA extraction

#### Study 2

Sham (*n* = 5) and TBI (*n* = 5) of C57BL/6J mice were anesthetized (100 mg/kg sodium pentobarbital, I.P.) at 7 days post-injury and transcardially perfused with ice-cold 0.9% saline (100 ml). Ipsilateral cortical and hippocampal tissues were rapidly dissected and processed for CD11b-positive selection and MP isolation.

### Enriched MP isolation and analysis

**Fig. 1:**
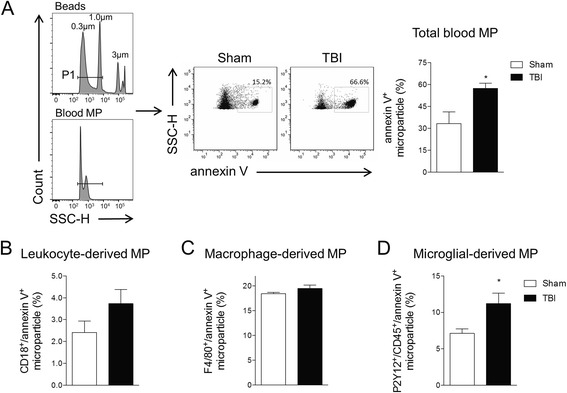
Microglial-derived MP are increased in the blood following TBI. Flow cytometry analysis of enriched MP in the blood from sham and TBI mice at 24 h post-injury. **a** Representation of gating strategy used to characterize MP using SSC-H and standard microbeads (300- to 1000-nm diameter). Standard microbeads (P1 gated population) were used as an internal control to determine the size of MP in the blood, and annexin V staining confirmed MP characteristics. At 24 h post-injury, total blood MP is increased in TBI mice compared with sham-injured mice. **b** Measurements of leukocyte-derived (CD18), macrophage-derived (F4/80), and microglial-derived (P2Y12/CD45) MP in the blood from sham and TBI mice at 24 h post-injury. Microglial-derived MP are significantly increased in TBI mice when compared with sham-injured mice (*p < 0.5 vs sham; Student’s *t* test; *n* = 6/group). *Bars* represent mean ± standard error of the mean (S.E.M.). Data represent results of three independent experiments

Mice were anesthetized (100 mg/kg sodium pentobarbital, I.P.), and blood was collected in heparinized syringes by aortic puncture. Blood was immediately combined with fixative (100 μl/ml Caltag Reagent A fixation medium, Invitrogen, Carlsbad, CA) to diminish ex vivo microparticle (MP) aggregation. All reagents and solutions used for MP analysis were sterile and filtered (0.1-μm filter). Heparinized blood was centrifuged for 5 min at 1500×*g*, and supernatant was centrifuged at 15,000×*g* for 30 min to pellet platelets and other cell debris [23]. Blood supernatants were used to collect total blood MP (~250 μl) that were purified by adding PBS (4 ml) and centrifuged at 100,000×*g* for 60 min at 4 °C as previously described [23–30]. In each experiment, flow cytometry in combination with microbead standards ranging in size from 300 to 3000 nm was used to characterize MP size. MP were distinguished from larger (apoptotic body; >1000 nm) and smaller (exosomes; <100 nm) vesicles based on size (SSC), and their phenotype was confirmed using the unique MP surface marker, annexin V (details below; see Fig. 1a). In parallel experiments, equal numbers of circulating MP from sham and TBI mice (8 × 10^5^ MP) were resuspended in 100 μl serum-free DMEM media and co-cultured with BV2 microglia (2 × 10^4^ cells/well) for 24 h at 37 °C and at 5% CO_2_ prior to cell extraction for markers of microglial activation. The enriched MP population obtained using this protocol may also contain other types of extracellular vesicles such as exosomes.

Total and cell-derived MP were identified by flow cytometry using an eight-color, triple-laser MACSQuant Analyzer (Miltenyi Biotec, Auburn, CA). MACSQuant was calibrated every other day with calibration beads (Miltenyi Biotec, Auburn, CA), and forward and side scatters were set at logarithmic gain. Photomultiplier tube voltage and triggers were optimized to detect submicron-sized particles. Microbeads of different diameters (0.3 μm (Sigma; LB3), 1.09 μm (Spherotech, Lake Forest, IL; BCP-10-5), and 3.0 μm (Spherotech, Lake Forest, IL; BP-30-5)) were used to set initial parameters and to confirm MP characteristics in each experiment. To reduce small particle contaminants, all reagents and solutions used for MP analysis were sterile and filtered (0.1-μm filter; EMD Millipore, Billerica, MA) before use. The expression of phosphatidylserine (PS) on MP was detected using an anti-annexin V (FITC or APC) (1:50, BD Biosciences PharMingen, catalog no: 556419) in annexin buffer (5 mM KCl, 1 mM MgCl_2_, 136 mM NaCl, 2 mM CaCl_2_, 1% BSA; pH 7.4). MP were incubated with antibodies for 30 min at room temperature (RT) in the dark. Annexin buffer (150 μl) was added to each sample prior to MACSQuant analysis. True-negative controls were established by a fluorescence-minus-one analysis and using isotype-matched irrelevant antibodies at the same concentration and under the same conditions. Annexin V-positive particles with diameters from 300 to 1000 nm were defined as MP. Blood MP were further characterized by double labeling with specific cell-specific antibodies: leukocytes (anti-CD18 (PE); 1:50, Biolegend; catalog no: 101408) and monocyte/macrophages (anti-F4/80 (APC); 1:50, Thermo Fisher, catalog no: MF48005). Microglial staining was performed using anti-P2Y12 (1:1000, AnaSpec Inc., Fremont, CA) and anti-CD45-PerCP (1:10, Miltenyi Biotec, Auburn, CA) as follows: MP were incubated with anti-P2Y12 in annexin buffer for 1 h at RT, washed and incubated with Alexa Fluor 488 goat anti-rabbit secondary antibodies (1:500; Life Technologies) for 30 min, washed and further incubated with pre-conjugated anti-CD45 for 30 min at RT, and washed in annexin buffer prior to analysis by flow cytometry. FlowJo software (Vx; Tree Star, Inc., Ashland, OR) was used for analysis, and data are presented as percent of annexin V-positive MP in the MP gate as set by microbeads, unless otherwise specified.

### In vitro MP analysis

BV2 microglia (murine microglial cell line) were cultured in DMEM (Invitrogen, Carlsbad, CA) supplemented with 10% fetal equine serum (HyClone, Logan, UT), and 1% penicillin and streptomycin (Invitrogen) at 37 °C with 5% CO_2_. DMEM media was filtered using 0.1-μm filters. BV2 microglia were seeded at a density of 0.6 × 10^6^ in 60-mm dishes and stimulated with lipopolysaccharide (LPS, 20 ng/ml; Sigma-Aldrich) or control media for 24 h. MP released into conditioned BV2 microglial media were characterized using a MACSQuant flow cytometer (Miltenyi Biotec, San Diego, CA) analysis as described before. In parallel experiments, MP in conditioned media were pelleted by centrifugation as described before and MP-enriched pellets were either resuspended in RIPA buffer (Teknova, Hollister, CA) for Western blotting or extracted using TRIzol reagent (Invitrogen) for RNA analysis. In another experiment, BV2 microglia cells were stained with 1 μM calcein AM (Invitrogen) in DMEM for 30 min at 37 °C and then stimulated with LPS (20 ng/ml) for 24 h. Calcein AM-labeled MP were isolated from conditioned media by centrifugation at 100,000×*g* for 60 min at 4 °C [23–30] and were stained with anti-annexin V (APC) (1:50, BD Biosciences PharMingen, catalog no: 550474) for MP characterization by flow cytometry as described before.

Levels of endotoxin (LPS) in pelleted MP from control and LPS-stimulated BV2 microglia were determined using a LAL Chromogenic Endotoxin assay (Thermo Fisher Scientific, MA, USA) as per manufacturer’s instructions. A standard curve was used to determine the concentration of endotoxin in each sample. Endotoxin levels are expressed as endotoxin unit per milliliter (EU/ml).

### MP co-culture studies

BV2 microglia were seeded at a density of 0.6 × 10^6^ in 60-mm dishes and stimulated with LPS (20 ng/ml) or control media for 24 h. MP released into conditioned BV2 microglial media were isolated by centrifugation as described before. Control and LPS-stimulated MP (total 8 × 10^5^ MP) were co-cultured with BV2 microglia (2 × 10^4^/96 well) or primary microglia (1 × 10^5^/96 well) that were obtained from postnatal day 1 C57BL/6 mouse pups as previously described [31] for 24 h at 37 °C and at 5% CO_2_ prior to cell extraction using TRIzol reagent (Invitrogen) to assess markers of microglial activation.

### MP isolation from CD11b-positive cells from the sham and TBI cortex

Magnetic bead-conjugated anti-CD11b and MACS separation technology (Miltenyi Biotec, Auburn, CA) was used to isolate microglia/macrophages from sham and TBI brain tissue of C57BL/6J mice at 7 days post-injury (*n* = 5/group; study 2 above). Briefly, ipsilateral cortical and hippocampal tissues were rapidly microdissected, and a single cell suspension was prepared using enzymatic digestion (Neural Tissue Dissociation Kit; Miltenyi Biotec) in combination with a gentle MACS dissociator. Myelin was removed using Myelin Removal Beads II and LS columns (Miltenyi Biotec), and the cells were incubated with anti-CD11b microbeads (Miltenyi Biotec) and loaded onto MS columns (Miltenyi Biotec) placed in the magnetic field of a MACS separator. The negative fraction (flow through) was collected, and the column was washed three times with MACS buffer (Miltenyi Biotech). CD11b-positive cells were eluted by removing the magnetic field, resulting in the isolation of approximately 93% viable CD11b-positive cells from sham and TBI mice [32]. Expression level of CD11b-positive selected cells (MFI) was quantified by flow cytometry using anti-CD11b (APC) (1:50; Miltenyi Biotech, catalog no: 130-091-241). CD11b-positive cells were subsequently seeded at 1 × 10^5^ cells/well in a 96-well plate in DMEM-F12 containing 10% fetal calf serum and incubated at 37 °C under 5% CO_2_ for 24 h. Ex vivo-secreted MP from sham and TBI CD11b-positive cells were collected from condition media by ultra centrifugation as described before.

### Intracortical injection of enriched MP

#### Study 1

Enriched MP were isolated from conditioned media of control and LPS (20 ng/ml)-stimulated BV2 microglia as described before. To neutralize the effects of MP, additional groups of control and LPS-stimulated MP were incubated with polyethylene glycol telomere B (PEG-TB; 6 μl per 100 μl media) for 1 h at RT as previously described [25]. MP were resuspended in 100 μl artificial CSF (Na 150, K 3.0, Ca 1.4, Mg 0.8, P 1.0, Cl 155 (mM); Harvard Apparatus, Holliston, MA, catalog no: 59-7316), and 1 μl (total 8 × 10^3^ MP) was injected intracortically into C57BL/6 mice (*n* = 6) at stereotactic coordinates of 2.0 mm anteroposterior, 1.0 mm mediolateral to the bregma, and 1.0 mm below the pia using a 33-gauge sharp needle attached to a 10-μl Hamilton syringe (Hamilton Medical, Reno, NV, USA) and an injection rate of 1 μl/10 min. The needle remained in situ for 5 min to prevent backflow before being withdrawn slowly over 10 min. Twenty-four hours later, the mice were euthanized and cortical tissue was collected using TRIzol reagent (Invitrogen) to assess markers of cortical neuroinflammation.

#### Study 2

MP were isolated from conditioned media of control and LPS (20 ng/ml)-stimulated BV2 microglia as described before, and 1 μl MP (total 8 × 10^3^ MP) were injected intracortically into C57BL/6 mice (*n* = 4) as described in study 1 above. Seven days later, mice were euthanized and the brains were removed to assess markers of microglial activation using histology.

#### Study 3

MP isolated from culture media of CD11b-positive cells from sham and TBI brain as described above were resuspended in 100 μl artificial CSF (Harvard Apparatus), and 1 μl MP (total 8 × 10^3^ MP) were injected intracortically into C57BL/6 mice (*n* = 4) as described above. Twenty-four hours later, the mice were euthanized and cortical tissue was collected using TRIzol reagent (Invitrogen) to assess markers of cortical neuroinflammation.

### Real-time PCR

Total RNA including miR was extracted from snap-frozen tissue, cells, or enriched MP, using a miRNeasy mini isolation kit (Qiagen, Valencia, CA). Complementary DNA (cDNA) synthesis was performed on 1 μg of total RNA using a Verso cDNA RT kit (Thermo Scientific, Pittsburg, PA), as per manufacturer’s instructions. For messenger RNA (mRNA) analysis, real-time PCR was performed using TaqMan gene expression assays on an ABI 7900 HT FAST Real-Time PCR machine (Applied Biosystems). Gene expression was calculated relative to the endogenous control sample (GAPDH) to determine relative expression values (2^−∆∆Ct^, where Ct is the threshold cycle). For miR analysis, a total of 10 ng of total RNA was reverse transcribed using TaqMan miRNA Reverse Transcription Kit (Applied Biosystems) with miR-specific primer of miR-155 and control U6. Reverse transcription reaction products (1.5 μl) were used for qPCR as described above. Following real-time PCR, miR expression was calculated relative to the endogenous control sample (U6) to determine relative expression values (2^−∆∆Ct^, where Ct is the threshold cycle).

### Western blotting

Proteins were extracted using RIPA buffer (Teknova, Hollister, CA), equalized, and loaded equally onto 5–20% gradient gels for SDS PAGE (Bio-Rad; Hercules, CA). Proteins were transferred onto nitrocellulose membranes and blocked overnight in 5% milk in 1x PBS containing 0.01% Tween-20 (PBS-T). The membrane was incubated in rabbit anti-interleukin-1β (anti-IL-1β) (1:1000; Cell Signaling, catalog no: sc-7884) and mouse anti-β-actin (1:20,000; Sigma, catalog no: A1978) overnight at 4 °C, then washed three times in PBS-T for 5 min, and incubated in appropriate HRP-conjugated secondary antibodies (Jackson Immuno Research Laboratories, West Grove, PA) for 1 h at RT. Membranes were washed three times in PBS-T, and proteins were visualized using Super Signal West Dura Extended Duration Substrate (Thermo Scientific, Rockford, IL). Chemiluminescence was captured using ChemiDoc TM XRS+ System (Bio-Rad; Hercules, CA), and protein bands were quantified by densitometric analysis using Image J (NIH, Bethesda, MD). The data reflects the intensity of the target protein band normalized based on the intensity of the endogenous control for each sample (expressed in arbitrary units). Protein from cells was normalized to β-actin, and protein from MP was normalized to Ponceau-S.

### Immunofluorescence imaging

Twenty-micrometer coronal brain sections from −1.70 mm from the bregma were selected, and standard immunostaining techniques were employed. Briefly, 20-μm sections were washed three times with 1x PBS, blocked for 1 h in goat serum containing 0.4% Triton X-100, and incubated overnight at 4 °C with primary antibody (rabbit anti-P2Y12 (1:1000, AnaSpec Inc., Fremont, CA; catalog no: AS-55043A), rabbit anti-Iba-1 (1:200; Wako Chemicals, Richmond, VA; catalog no: 019-197)). Sections were washed three times with 1x PBS and incubated with appropriate Alexa Fluor-conjugated secondary antibodies (Life Technologies) for 2 h at RT. Sections were washed three times with 1x PBS, counterstained with 4′,6-diamidino-2-phenylindole (DAPI; 1 μg/ml, Sigma), and mounted with glass cover slips using Hydromount solution (National Diagnostics, Atlanta, GA). Images were acquired using a fluorescent Nikon Ti-E inverted microscope, at ×10 (Plan Apo 10× NA 0.45) or ×20 (Plan APO 20× NA 0.75) magnification. Exposure times were kept constant for all sections in each experiment. All images were quantified using Nikon ND-Elements Software (AR 4.20.01). 6000–10,000 positive areas were quantified per mouse per experiment, and expression levels were expressed as binary area per region of interest (ROI) × 10^6^.

### Assessment of microglial morphology

Neurolucida software (MBF Biosciences, Williston, VT) was used to quantify cell body area and ramification length of P2Y12-positive microglia in the cortex, hippocampus, and thalamus of enriched MP intracortical injected mice as previously described [33]. Immunostained microglia were outlined using the live image setting so that the width of the ramified branches could be traced while focusing on the section. Cell bodies were outlined using the contour tool followed by tracing of the individual ramification using the dendrite line tool. Microglial ramification length is expressed in micrometers,, and cell body area is expressed in square millimeters.

### Statistical analysis

Randomization and blinding protocols were employed, and individuals performing in vitro and in vivo analysis were blinded to isolated MP groups. Quantitative data were expressed as mean ± standard errors of the mean (S.E.M.). qPCR and flow cytometry data were analyzed by one-way ANOVA, followed by post hoc adjustments using a Student-Newman-Keuls test. Remaining data were analyzed using Student’s *t* test. Statistical analyses were performed using GraphPad Prism Program, Version 3.02 for Windows (GraphPad Software, San Diego, CA, USA). A *p* < 0.05 was considered statistically significant.

## Results

### Microglia-derived microparticles are released into circulation following TBI

C57BL/6 male mice were subjected to TBI (moderate-level CCI) or sham surgery, and 24 h later, animals were euthanized and blood was collected for MP isolation and characterization. Ipsilateral cortical tissue was also collected to assess markers of brain inflammation. MP in blood were measured by flow cytometry for annexin V, which binds the externalized phosphatidylserine (PS) present on the surface of MP. To exclude the presence of annexin V-positive apoptotic bodies (>1000 nm) and smaller exosomes (<100 nm) in the sample, microbead standards (300, 1000, and 3000 nm) were used to gate on MP (MP gate set between 300 and 1000 nm) (Fig. 1a). There was a significant increase in annexin V-positive MP in the blood of TBI mice when compared to sham-injured controls (*p* < 0.05; Fig. 1a).

We next evaluated the origin of the circulating MP based on unique markers derived from parental cells. P2Y12/CD45-positive MP (microglial derived) were significantly increased in the circulation when compared to sham-injured control levels (*p* < 0.05; Fig. 1b). The CD18-positive MP (leukocyte derived) increase after TBI did not reach statistical significance, and TBI did not increase levels of F4/80-positive MP (monocyte derived) in the circulation at 24 h post-injury. These data indicate that microglial-derived MP are released into the circulation after moderate-level TBI.

**Fig. 2:**
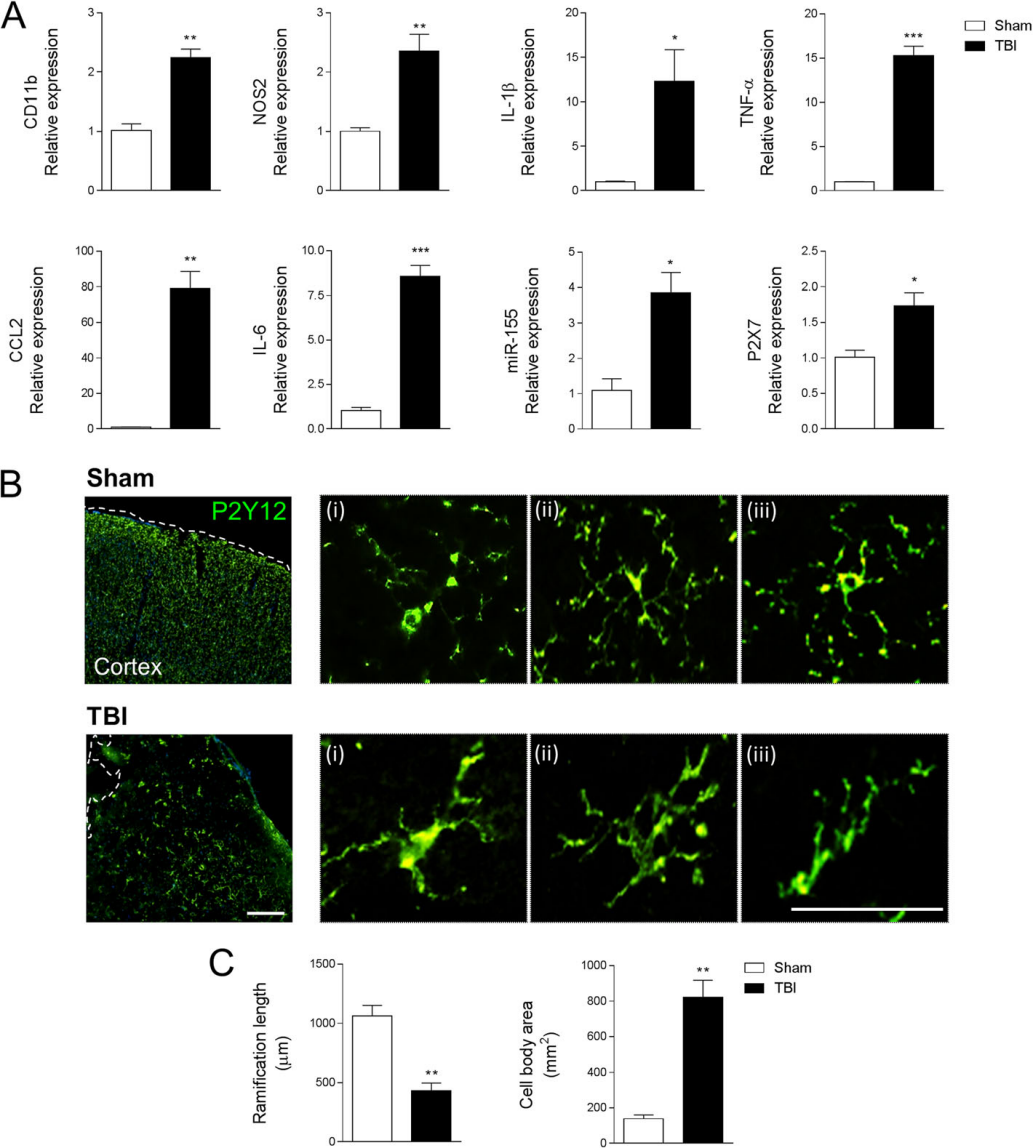
Microglial activation in the TBI brain at 24 h post-injury. **a** Gene expression analysis of microglia activation in the cortex of sham and TBI mice at 24 h post-injury. Microglial receptors (CD11b, P2X7) and pro-inflammatory mediators (NOS2, IL-1β, TNF-α, CCL2, IL-6, and miR-155) were significantly increased in the injured cortex at 24 h post-injury (*p < 0.05, **p < 0.01, and ***p < 0.001 vs sham-injured; Student’s *t* test; *n* = 6/group). **b** Immunofluorescence imaging for P2Y12-positive microglia in the cortex of sham and TBI mice at 24 h post-injury. Following TBI P2Y12-positive microglia transformed from a ramified morphology in sham to activated morphology displaying enlarged cell body, and thicker, and shorter, projections. Representative images taken at −2.06 mm from the bregma. *Scale bar* = 50 μm. **c** Morphological analysis of P2Y12-positive microglia using 3D-reconstruction Neurolucida software. When compared to sham-injured controls, P2Y12-positive microglia in the TBI cortex had reduced ramification length (**p < 0.01; Student’s *t* test) and an enlarged cell body area (**p < 0.01; Student’s *t* test; *n* = 6/group). *Bars* represent mean ± S.E.M.

Assessment of injured cortical tissue revealed a robust neuroinflammatory response to TBI, with increased mRNA expression of markers of microglial activation and the induction of pro-inflammatory cytokines and chemokines. Specifically, TBI resulted in a significant increase in mRNA for CD11b (*p* < 0.01), nitric oxide synthase 2 (NOS2, p < 0.01), interleukin-1β (IL-1β, *p* < 0.05), tumor necrosis factor-alpha (TNF-α, p < 0.001), C-C motif chemokine ligand 2 (CCL2, p < 0.01), interleukin-6 (IL-6, *p* < 0.001), pro-inflammatory microRNA-155 (miR-155, *p* < 0.05), and the purinergic receptor P2X7 (*p* < 0.05) when compared to the sham-injured group (Fig. 2a). Histological assessment of P2Y12 expression on microglia in the ipsilateral cortex revealed that the following moderate TBI P2Y12-positive microglia transform from a ramified morphological state to an activated state that withdrew branched processes to form thick bundles around highly enlarged cell bodies (Fig. 2b). Further, Neurolucida reconstruction analysis of P2Y12-positive cells demonstrated that ramification length was significantly decreased (*p* < 0.01) and cell body area was significantly increased (*p* < 0.01) in microglia in the TBI cortex as compared to sham-injured cortex (Fig. 2c).

### TBI-induced circulating microparticles activate microglia in vitro

**Fig. 3:**
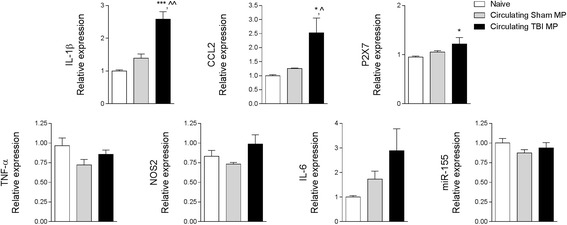
Circulating blood MP released after TBI activate naive BV2 microglia. Enriched MP were isolated from the blood of sham-injured and TBI mice and were co-cultured with naïve BV2 microglia cells for 24 h. Microglial receptors (P2X7) and pro-inflammatory mediators (IL-1β and CCL2) were significantly increased in BV2 microglia treated with circulating TBI MP (*p < 0.05 and ***p < 0.001 vs naïve; ^*p* < 0.05 and ^^*p* < 0.01 vs sham MP; one-way ANOVA with Student-Newman-Keuls correction for multiple comparisons; *n* = 6/group). There were no significant differences in TNF-α, NOS2, IL-6, and miR-155 expression between treatment groups. *Bars* represent mean ± S.E.M.

To examine if circulating MP released after TBI could promote inflammation, we co-cultured BV2 microglia in the presence or absence of enriched MP from the blood of sham-injured and TBI mice (Fig. 3). Equal number of circulating enriched MP from sham and TBI blood (total 8 × 10^5^) were incubated with naive BV2 microglia for 24 h, and subsequent activation was assessed by mRNA expression of selected microglial activation markers. When compared to BV2 microglia treated with sham circulating enriched MP, there was a significant increase in IL-1β (*p* < 0.01) and CCL2 (*p* < 0.05) in recipient microglia co-cultured with TBI circulating enriched MP (Fig. 3) and a trend towards increased in IL-6 and NOS2 expression in this group. There was also a significant increase in P2X7 (*p* < 0.05) in recipient microglia treated with TBI circulating enriched MP when compared to naïve microglia. There was no difference in expression of other pro-inflammatory molecules such as TNF-α and miR-155.

### LPS stimulation increases microparticle release in BV2 microglia, and pro-inflammatory molecules are enriched in microparticles

**Fig. 4:**
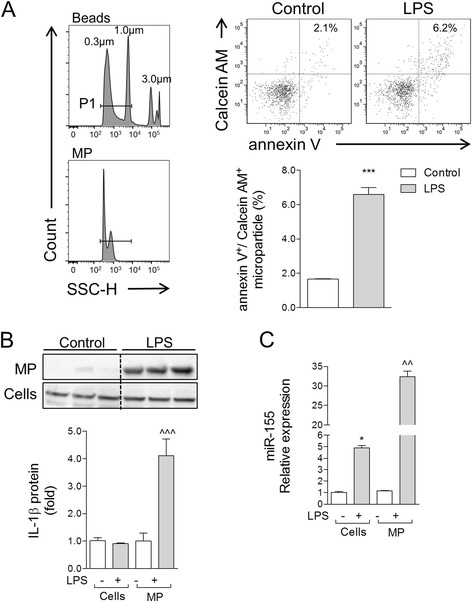
Pro-inflammatory mediators are enriched in MP following lipopolysaccharide stimulation of BV2 microglia. **a** Calcein AM-stained BV2 microglia were stimulated with LPS (20 ng/ml) for 24 h, and MP were isolated by differential centrifugation and stained with anti-annexin V, prior to characterization by flow cytometry. When compared to MP levels in control BV2 microglia, LPS stimulation significantly increased the number of annexin V/calcein AM-positive MP (***p < 0.001 vs control; Student’s *t* test; *n* = 4/group). **b** IL-1β protein expression in enriched MP vs cell lysates of control and LPS-stimulated BV2 microglia. Western blot analysis demonstrated that IL-1β protein was significantly increased in enriched MP following LPS stimulation (^^^*p* < 0.001 vs control MP; one-way ANOVA with Student-Newman-Keuls correction for multiple comparisons; *n* = 3/group). Lanes 1, 2, and 3 in both control and LPS refer to sample replicates. **c** miR-155 expression in enriched MP vs cell lysates of control and LPS-stimulated BV2 microglia. miR-155 was significantly increased in cell lysates of BV2 microglia following LPS stimulation (*p < 0.05 vs control cells), and its expression was elevated further in enriched MP (^^*p* < 0.01 vs control MP; one-way ANOVA with Student-Newman-Keuls correction for multiple comparisons; *n* = 4/group). *Bars* represent mean ± S.E.M. Data represent results of three independent experiments

We performed a MP release assay in BV2 microglia following LPS stimulation (20 ng/ml) for 24 h. MP release was quantified by flow cytometry using Calcein AM-stained BV2 microglia that were co-stained with the MP marker annexin V. When compared to MP levels in control BV2 microglia, LPS stimulation significantly increased the number of annexin V/calcein AM-positive MP (*p* < 0.001 vs control; Fig. 4a). We next measured IL-1β and miR-155 in BV2 microglial cells and isolated enriched MP from the conditioned media to determine the relative expression of pro-inflammatory mediators in MP vs cellular compartments. Following LPS stimulation, IL-1β protein was not detected in the cells, but it was significantly increased in LPS-stimulated enriched MP (*p* < 0.001 vs LPS-stimulated cells; Fig. 4b). In addition, following LPS stimulation, miR-155 was significantly increased in BV2 microglial cells (*p* < 0.05 vs control cells; Fig. 4c). Notably, miR-155 was even more highly enriched in LPS-stimulated MP (*p* < 0.01 vs LPS-stimulated cells), indicating that enriched MP contain elevated concentrations of pro-inflammatory molecules IL-1β and miR-155 following activation.

### LPS-stimulated microparticles activate microglia in vitro

**Fig. 5:**
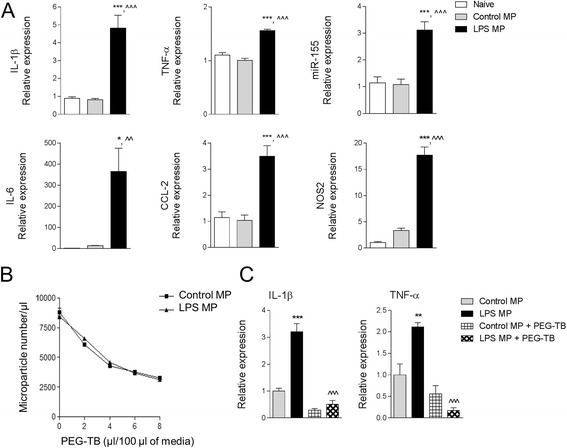
Lipopolysaccharide-stimulated MP activate BV2 microglia. **a** Enriched MP were isolated from control and LPS-stimulated BV2 microglia and were co-cultured with naïve BV2 microglia for 24 h. Pro-inflammatory mediators (IL-1β, TNF-α, miR-155, IL-6, CCL2, and NOS2) were significantly increased in BV2 microglia treated with LPS MP (*p < 0.05 and ***p < 0.001 vs naïve; ^^*p* < 0.01 and ^^^*p* < 0.001 vs control MP; one-way ANOVA with Student-Newman-Keuls correction for multiple comparisons; *n* = 4/group). Data represent results of three independent experiments. **b** MP neutralization using PEG-TB. Enriched MP from control and LPS-stimulated BV2 microglia were incubated with increasing concentrations of PEG-TB for 1 h, and number of MP were quantified by flow cytometry. 6 μl PEG-TB/100 μl resulted in significant depletion of MP under both conditions. **c** Naïve BV2 microglia were co-cultured with control or LPS-stimulated MP ± PEG-TB (6 μl/100 μl) for 24 h. LPS MP treatment increased IL-1β and TNF-α in BV2 microglia (**p < 0.01 and ***p < 0.001 vs control MP), whereas co-treatment with PEG-TBI resulted in a significant decrease in IL-1β and TNF-α expression (^^^p < 0.001 vs LPS MP; one-way ANOVA with Student-Newman-Keuls correction for multiple comparisons; *n* = 6/group). *Bars* represent mean ± S.E.M.

To test the hypothesis that enriched MP from activated microglia can seed neuroinflammation, we isolated MP from control and LPS-stimulated BV2 microglia and co-cultured them with naïve BV2 or primary microglia for 24 h prior to assessing cellular markers of activation by qPCR. When compared to control enriched-MP-treated recipient BV2 microglia, there was a significant increase in IL-1β (*p* < 0.001), TNF-α (*p* < 0.001), CCL2 (*p* < 0.001), IL-6 (*p* < 0.01), and NOS2 (*p* < 0.001) mRNA in recipient BV2 microglia treated with LPS enriched MP (Fig. 5a). There was also a significant increase in miR-155 expression in BV2 microglia (*p* < 0.001) treated with LPS enriched MP when compared to cells treated with control MP (Fig. 5a).

**Fig. 6:**
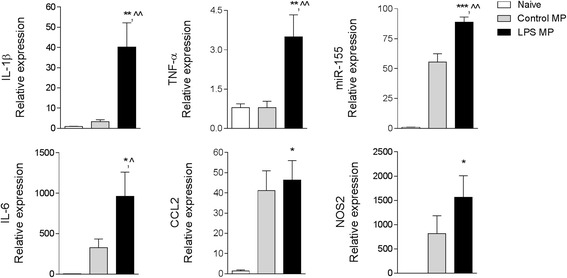
Lipopolysaccharide-stimulated MP activate primary cortical microglia. Enriched MP were isolated from control and LPS-stimulated BV2 microglia and were co-cultured with primary cortical microglia for 24 h. Pro-inflammatory mediators (IL-1β, TNF-α, miR-155, IL-6, CCL2, and NOS2) were significantly increased in primary microglia treated with LPS MP (*p < 0.05, **p < 0.001, and ***p < 0.001 vs naïve; ^*p* < 0.05 and ^^*p* < 0.01 vs control MP; one-way ANOVA with Student-Newman-Keuls correction for multiple comparisons; *n* = 5/group). *Bars* represent mean ± S.E.M.

To demonstrate the specific activity of microglial-derived enriched MP in activating microglia in these experiments, we neutralized MP by co-incubating them with polyethylene glycol telomere B (PEG-TB), a surfactant that depletes MP without activating blood immune cells [25]. To establish the dose of PEG-TB required to neutralize microglial-derived MP, we performed a dose response study in control and LPS-stimulated enriched MP derived from BV2 microglia and incubated them with increasing concentrations of PEG-TB for 1 h prior to MP characterization by flow cytometry. We determined that 6 μl PEG-TB/100 μl significantly depleted MP levels under both conditions (Fig. 5b), and this concentration was employed to neutralize microglial-derived MP activity in in vitro studies. Next, we determined that when LPS enriched MP were neutralized by PEG-TB and co-cultured with naïve BV2 microglia for 24 h, markers of microglial activation were significantly reduced. Specifically, recipient BV2 microglia incubated with LPS MP + PEG-TB had significantly reduced expression of IL-1β (*p* < 0.001) and TNF-α (*p* < 0.001) when compared to the LPS MP group (Fig. 5c). We confirmed the effects of LPS-stimulated enriched MP on primary cortical microglia. There was a significant increase in IL-1β (*p* < 0.01), TNF-α (*p* < 0.01), and IL-6 (*p* < 0.05) mRNA in recipient primary microglia treated with LPS enriched MP when compared to control MP-treated cells (Fig. 6). There was also a significant increase in miR-155 expression in primary microglia (*p* < 0.01) treated with LPS enriched MP when compared to cells treated with control MP (Fig. 6).

Finally, we confirmed that the effects of LPS enriched MP on microglial activation were not due to LPS crossover. We measured LPS levels in the LPS enriched MP samples using a limulus amebocyte lysate (LAL) assay and determined that the LPS concentration was negligible (0.081 ± 0.009 EU/ml (<0.1 ng)) and well below LPS concentrations previously shown to transfer LPS activity in enriched MP [34].

### Microglial-derived microparticles can seed brain inflammation in vivo

**Fig. 7:**
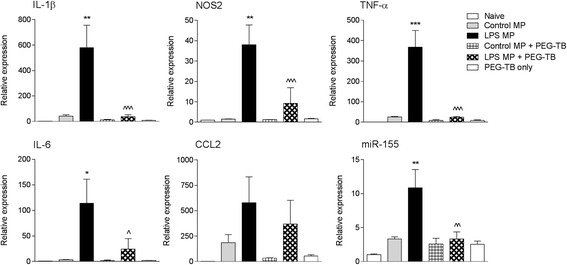
Lipopolysaccharide-stimulated MP increase neuroinflammation in the cortex of uninjured mice. Enriched MP were isolated from control and LPS-stimulated BV2 microglia and were treated ± PEG-TB (6 μl/100 μl) prior to being stereotactically injected into the cortex of adult male C57BL/6 mice. Markers of cortical neuroinflammation were measured at 24 h postinjection. There was a significant increase in pro-inflammatory mediators (IL-1β, TNF-α, miR-155, IL-6, and NOS2) in the cortex of LPS MP-injected mice (**p < 0.01, ***p < 0.001 vs control MP-injected group). Neutralization of LPS MP prior to injection resulted in a significant decrease in each pro-inflammatory mediator (^*p* < 0.05, ^^*p* < 0.01, ^^^*p* < 0.001 vs LPS MP-injected group; one-way ANOVA with Student-Newman-Keuls correction for multiple comparisons; *n* = 6/group). *Bars* represent mean ± S.E.M.

To determine whether microglial-derived enriched MP could seed neuroinflammation in the uninjured brain, we isolated enriched MP from control and LPS-stimulated BV2 microglia and stereotactically injected them into the cortex of adult male C57BL/6 mice. As a control to demonstrate the specific activity of microglial-derived enriched MP in promoting brain inflammation, we neutralized enriched MP by co-incubating them with PEG-TB as described before. After 24 h, cortical tissue was collected and markers of brain inflammation were assessed. When LPS enriched MP were injected into the uninjured cortex, they produced a robust neuroinflammatory response resulting in a significant increase in IL-1β (*p* < 0.01), NOS2 (*p* < 0.01), TNF-α (*p* < 0.001), IL-6 (*p* < 0.05), and miR-155 (*p* < 0.01) when compared to the control MP-injected group (Fig. 7). Notably, when LPS enriched MP were neutralized by PEG-TB and injected into the uninjured cortex, the cortical neuroinflammatory response was attenuated, such that the LPS enriched MP + PEG-TB group had significantly reduced expression of IL-1β (*p* < 0.001), NOS2 (*p* < 0.001), TNF-α (*p* < 0.001), IL-6 (*p* < 0.05), and miR-155 (*p* < 0.01) when compared to the LPS enriched MP group (Fig. 7). Intracortical injection with control MP, control MP + PEG-TB, or PEG-TB alone did not promote a neuroinflammatory response in the cortex.

**Fig. 8:**
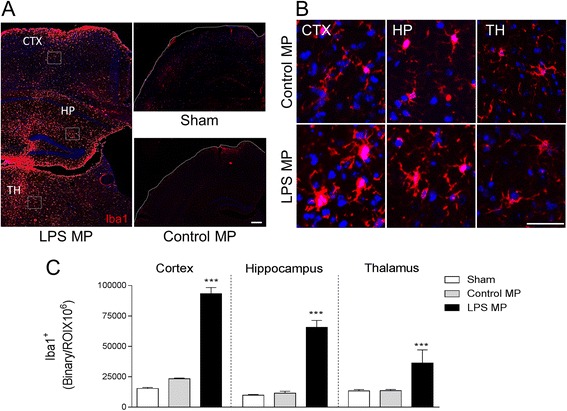
Lipopolysaccharide-stimulated MP increase microglial activation in the cortex of uninjured mice. Enriched MP were isolated from control and LPS-stimulated BV2 microglia and were stereotactically injected into the cortex of adult male C57BL/6 mice. Iba-1 immunocytochemistry was performed at 7 days postinjection. **a** Representative Iba-1 staining (*red*) in the cortex (*CTX*), hippocampus (*HP*), and thalamus (*TH*). Images taken at −2.06 mm from the bregma; *scale bar* = 50 μm. **b** High-magnification images in control MP- and LPS MP-injected mice in the *CTX*, *HP*, and *TH*. LPS MP-injected Iba-1-positive microglia had enlarged cell body and thicker projection indicative of increased activation status. *Scale bar* = 100 μm. **c** Quantification of Iba-1 staining in the cortex, hippocampus, and thalamus at 7 days postinjection. There was a significant increase in Iba-1 immunoreactivity in the LPS MP-injected group when compared to the control MP-treated group (***p < 0.001 vs control MP; one-way ANOVA with Student-Newman-Keuls correction for multiple comparisons; *n* = 4/group). *Bars* represent mean ± S.E.M.

**Fig. 9:**
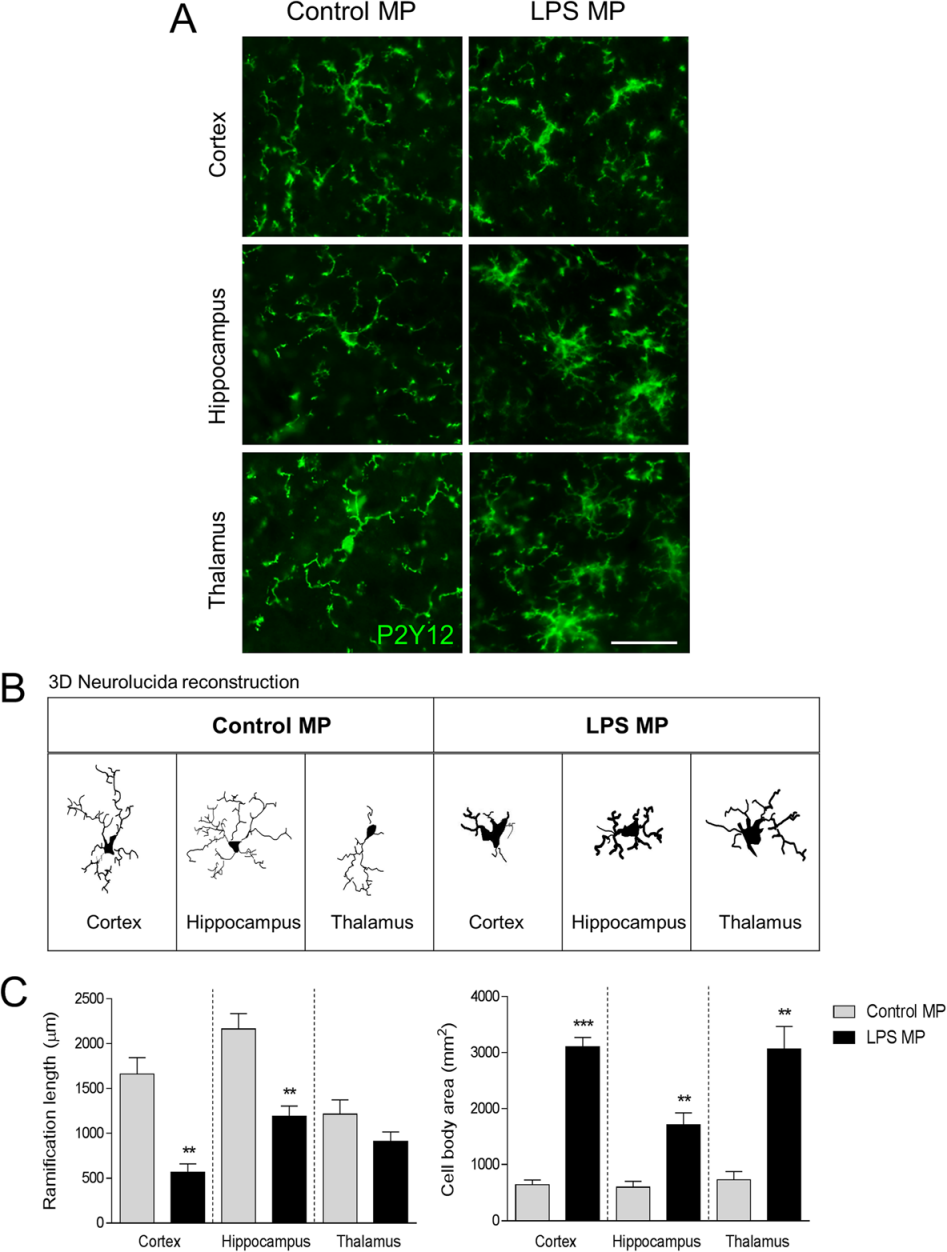
Lipopolysaccharide-stimulated MP alter P2Y12 microglial morphology of the cortex of uninjured mice. Enriched MP were isolated from control and LPS-stimulated BV2 microglia and were stereotactically injected into the cortex of adult male C57BL/6 mice. P2Y12 immunocytochemistry was performed at 7 days postinjection. **a** High-magnification images of P2Y12-positive microglia (*green*) in control MP- and LPS MP-injected mice in the cortex (CTX), hippocampus (HP), and thalamus (TH). LPS MP-injected P2Y12-positive microglia have enlarged cell body and thicker projection indicative of increased activation status. *Scale bar* = 100 μm. **b** P2Y12-positive microglia in the CTX, HP, and TH of control MP- and LPS MP-injected mice. **c** Morphological analysis of P2Y12-positive microglia using 3D-reconstruction Neurolucida software. When compared to the control MP-injected group, P2Y12-positive microglia in the LPS MP-injected group had reduced ramification length in the cortex and hippocampus (**p < 0.01; Student’s *t* test), but not in the thalamus. In addition, the LPS MP-injected group had enlarged cell body area in each region (**p < 0.01 and ***p < 0.001 vs control MP; Student’s *t* test; *n* = 4/group). *Bars* represent mean ± S.E.M.

In a separate study, control and LPS enriched MP were stereotactically injected into the cortex of uninjured C57BL/6 mice. At 7 days post-surgery, mice were euthanized and brain tissue was saline perfused and fixed for immunohistochemical analysis of microglial activation in the cortex, hippocampus, and thalamus using Iba-1 and P2Y12 immunostaining. There was a significant increase in Iba-1 staining in the ipsilateral cortex, hippocampus, and thalamus of C57BL/6 injected with LPS enriched MP (*p* < 0.001) when compared to mice injected with control MP or sham mice (Fig. 8a–c). Further morphological analysis of P2Y12-positive microglia using Neurolucida reconstruction software revealed that C57BL/6 mice injected with LPS enriched MP resulted in increased microglial activation. Specifically, microglial ramifications were significantly reduced in length in the cortex and hippocampus of the LPS enriched-MP-injected cortex (*p* < 0.01 for both vs control MP), and the microglial cell body area was significantly increased in the cortex (*p* < 0.001), hippocampus (*p* < 0.01), and thalamus (*p* < 0.01 vs control MP; Fig. 9a–c). These data indicate that enriched MP derived from LPS-stimulated microglia produce a robust neuroinflammatory response when injected into the cortex of uninjured mice.

### Microparticles produced by microglia/macrophage isolated from the TBI brain induce neuroinflammation in non-injured mice

**Fig. 10:**
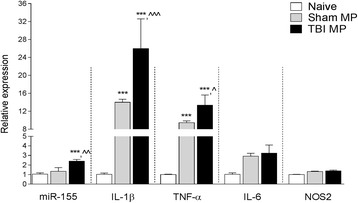
MP isolated from CD11b-isolated microglia/macrophages following TBI increase neuroinflammation in the cortex of uninjured mice. CD11b-microglia/macrophages in the cortex of sham and TBI were isolated at 7 days post-injury and cultured for 24 h prior to collecting MP. Enriched MP were stereotactically injected into the cortex of adult male C57BL/6 mice and markers of cortical neuroinflammation were measured at 24 h postinjection. There was a significant increase in pro-inflammatory mediators (IL-1β and TNF-α) in the cortex of the control MP-injected group (***p < 0.001 vs naïve group). When compared to the control MP-injected group, there was a further significant increase in pro-inflammatory mediators (IL-1β, TNF-α, and miR-155) in the cortex of the TBI MP-injected group (^*p* < 0.05, ^^*p* < 0.01, ^^^*p* < 0.001 vs control MP-injected group; one-way ANOVA with Student-Newman-Keuls correction for multiple comparisons; *n* = 5/group). *Bars* represent mean ± S.E.M.

To relate MP-mediated seeding of neuroinflammation to secondary injury mechanisms in the TBI brain, we subjected adult male C57BL/6 mice to sham or moderate-level TBI and isolated microglia/macrophages from the ipsilateral cortex at 7 days post-injury using MACS CD11b magnetic beads. We then cultured CD11b-positive cells for 24 h and collected microglia/macrophage-derived enriched MP from sham and TBI samples. Equal numbers of enriched MP (total 8 × 10^3^ MP) were stereotactically injected into the cortex of uninjured adult male C57BL/6 mice, and cortical tissue was collected 24 h later to assess markers of neuroinflammation. Injection of sham MP significantly increased IL-1β (*p* < 0.001) and TNF-α (*p* < 0.001) expression, but not miR-155 expression, when compared to the naïve control group (Fig. 10). Furthermore, injection of TBI enriched MP significantly increased IL-1β (*p* < 0.001), TNF-α (*p* < 0.05), and miR-155 (*p* < 0.01) expression in the cortex when compared to the sham MP-injected group. Although there was a modest increase in IL-6 and NOS2 expression in sham MP and TBI enriched MP-injected groups when compared to the naïve control group, these changes did not reach statistical significance. These data indicate that enriched MP derived from the microglia/macrophages isolated from the TBI brain produce a robust neuroinflammatory response when injected into the uninjured cortex.

## Discussion

TBI initiates complex local and systemic immune responses. However, the precise nature of posttraumatic neuroinflammation, its regulatory mechanisms, and role in secondary injury remain to be elucidated [35]. Recent studies have offered intriguing evidence regarding the potential role played by extracellular vesicles in cell-cell communication between immune cells and their targets [36, 37]. In the present study, we examined the release of microparticles (MP), a special class of extracellular vesicles, from the injured brain and their contribution to mechanisms of microglial activation and related neuroinflammation.

Although high levels of MP have been detected in the blood of TBI patients [18, 19], their cellular origins are not well defined. Clinical studies indicate that MP in the blood are primarily derived from platelets, with much smaller fractions released from erythrocytes, granulocytes, monocytes, lymphocytes, and endothelial cells [18, 19, 21, 38, 39]. Recent studies have highlighted the potential systemic pathological effects of MP following brain injury, including brain-trauma-associated coagulopathy [21]. MP released from neurons (NSE-positive MP) and astrocytes (GFAP-positive MP) accumulated in the circulation within 3 h of experimental TBI and were associated with microvascular fibrin deposition in the heart, kidney, and lung [21]. We observed a significant increase in total circulating MP at 24 h post-injury. Furthermore, an evaluation of the origin of circulating MP based on the presence of unique markers derived from parental cells revealed that microglial MP displayed the greatest increase (approximately twofold) following TBI, accounting for nearly 15% of total circulating MP. Although P2Y12 is also expressed on platelets [40], the detected P2Y12-positive MP were co-stained with CD45, a unique myeloid cell marker that is not expressed on platelets [41, 42], indicating that the P2Y12-positive MP enriched in the circulation following TBI were not derived from platelets. These data indicate that microglial-derived MP are released by the TBI brain and reach the systemic circulation. Importantly, we observed robust neuroinflammatory responses in the injured brain—with activation of P2Y12-positive microglia in the cortex, hippocampus, and thalamus—associated with up-regulated expression of classical pro-inflammatory mediators (IL-1β, TNF-α, CCL2, IL-6, NOS2, and miR-155) in the injured cortex.

Previous studies indicated that microglia and other myeloid cells in vitro can shed MP, which store and release pro-inflammatory molecules such as IL-1β [43], inflammasome components, and MHCII protein [44]. These data suggest that MP produced by reactive myeloid cells, such as microglia, may propagate inflammation and the rapid dissemination and presentation of antigens. A major finding of our study was that circulating enriched MP from TBI mice significantly activated recipient microglia in vitro and up-regulated pro-inflammatory signaling molecules such as IL-1β and CCL2.

Similarly, MP from LPS-stimulated BV2 microglia significantly increased IL-1β, TNF-α, CCL2, IL-6, NOS2, and miR-155 expression in recipient BV2 or primary microglia, confirming the ability of MP to act as independent microglial activators. LPS stimulation of microglia is an established in vitro model for TBI neuroinflammation because LPS up-regulates key pro-inflammatory mediators in microglia (IL-1β, TNF-α, NOS2, CCL2) that are robustly up-regulated in microglia/macrophages in the injured cortex and hippocampus following TBI [31, 45, 46]. In the current study, we demonstrated that LPS-stimulated microglia release MP into conditioned media that can induce pro-inflammatory responses in non-activated recipient microglial cells. Importantly, MP depleted of their content by addition of MP-neutralizing surfactant, PEG-TB, lost their ability to activate recipient cells, thus demonstrating the critical function of MP in promoting the pro-inflammatory response in the target cells.

We demonstrated that IL-1β and miR-155 were highly enriched in MP that can propagate a pro-inflammatory response in recipient cells. IL-1β does not contain an N-terminal signal sequence for secretion and therefore must be released from the cell through alternative mechanisms [47]. LPS stimulation increased IL-1β protein in enriched MP but not in microglia cells, where only IL-1β mRNA was elevated. Astrocyte-derived ATP has been shown to induce extracellular vesicle shedding and IL-1β release in microglia through a P2X7 receptor-dependent mechanism [43]. Notably, ATP is released following acute brain injury and promotes a powerful chemotactic response in microglia towards the site of injury [15]. In our study, the P2X7 receptor was significantly increased at sites of inflammation in the injured cortex; thus, P2X7 receptor-dependent mechanisms of MP release in microglia may be involved in the propagation of inflammation following TBI. Other mechanisms of MP release in microglia—such as MP fusion with the cell membrane, macropinocytosis [48], and direct release of their contents into the cytosol [37], as well as indirect mechanisms through binding of pattern-recognition receptors in the endosomal compartment (primarily Toll-like receptor 7/8—TLR7/8) [49]—may also contribute to the propagating neuroinflammation and warrant further investigation.

MP can also transfer miRs [50–53]. The levels of miR-155, a well-characterized pro-inflammatory miR in microglia [54], were highly enriched in microglial-derived MP. miR-155 has been shown to be a key regulator of the inflammatory response in experimental models of stroke, Parkinson’s disease, amyotrophic lateral sclerosis, and multiple sclerosis [55–58]. We also demonstrated that miR-155 was significantly increased in the cortex of TBI mice, and its expression was significantly increased when LPS-stimulated MP from BV2 microglia or MP from CD11b-positive microglia/macrophages from the TBI brain were injected into the cortex of uninjured mice. Secreted miR-155 from adipocyte-derived MP in obese mice induces a pro-inflammatory activation state in macrophages that causes chronic inflammation and local insulin resistance [59]. Thus, secreted miR-155 from MP may be an important driver of neuroinflammation following TBI.

The pathogenic role of MP in the inflammatory response was demonstrated in vivo by showing that injection of microglial-derived MP induces neuroinflammation at the site of injection and at more distant sites. Cortical injection of enriched MP isolated from LPS-stimulated BV2 microglia significantly increased markers of microglial activation (Iba-1 and P2Y12 morphological transformation) in the ipsilateral cortex, hippocampus, and thalamus and up-regulated pro-inflammatory markers in the cortex. These data support prior research that demonstrated that myeloid-derived microvesicles that are detected in the CSF of multiple-sclerosis patients and closely associate with disease course can propagate inflammation in vivo when injected locally [60]. Furthermore, cortical injections of enriched MP collected from ex vivo cultures of microglia/macrophages purified from TBI brain markedly induce expression of pro-inflammatory molecules miR-155, IL-1β, and TNF-α in the cortex of non-injured animals. To our knowledge, these latter observations describe the first use of purified brain microglia to demonstrate the transfer of the posttraumatic neuroinflammatory phenotype using MP as a vehicle.

It is important to recognize that the enriched MP population obtained using our experimental protocol may also contain other types of extracellular vesicles such as exosomes. In this study, we used established flow cytometry protocols to characterize MP properties [25], but this technique is limited to the identification of particles greater than 300 nm, preventing the detection of smaller microvesicles and all exosomes [61]. Electron microscopy can directly show that extracellular vesicles exist in a sample, but fixation processes involved in the technique can alter vesicle shape and size [62]. Other techniques such as dynamic light scattering and nanoparticle-tracking analyses have several limitations and introduce biases when characterizing extracellular vesicle properties [61, 63, 64]. The focus of the current study was the pathophysiological responses of microglial-derived MP rather than the nature of the enriched microvesicles. We determined that enriched MP derived from microglia could propagate neuroinflammation in vivo. We selectively depleted enriched MP by incubating them with PEG-TB, a drug that emulsifies MP without modifying circulating leukocyte activation [2, 25]. When we co-cultured depleted MP from LPS-stimulated BV2 microglia with recipient naïve BV2 microglia, or injected depleted MP into the cortex of uninjured mice, pro-inflammatory responses were significantly attenuated. These results support our hypothesis that it is the enriched MP component of purified extracellular vesicles derived from microglia that propagates neuroinflammation.

## Conclusions

The major findings of these studies are that (1) microglial-derived MP are released after TBI, (2) circulating enriched MP from the TBI animals can activate microglia in vitro, (3) LPS activation increases MP release from microglia and elevates their content of pro-inflammatory mediators IL-1β and miR-155, and (4) enriched MP from activated microglia in vitro or CD11b-isolated microglia from the TBI brain ex vivo are sufficient to initiate neuroinflammation following intracortical injection in naïve animals. Given their ability to independently initiate pro-inflammatory responses, MP derived from activated microglia may provide a novel therapeutic target for TBI and other neurodegenerative disorders associated with neuroinflammation.

## Abbreviations

CCI: Controlled cortical impact
CCL2: C-C motif chemokine ligand 2
IL-1β: Interleukin-1β
IL-6: Interleukin-6
miR-155: MicroRNA-155
MP: Microparticles
NOS2: Nitric oxide synthase 2
P2X7: P2X purinoceptor 7
PEG-TB: Polyethylene glycol telomere B
PS: Phosphatidylserine
SOD1: Superoxide dismutase 1
TBI: Traumatic brain injury
TNF-α: Tumor necrosis factor-alpha
